# Waves of family hope: narratives of families in the context of pediatric chronic illness*

**DOI:** 10.1590/1518-8345.5515.3504

**Published:** 2021-11-19

**Authors:** Ana Carolina Andrade Biaggi Leite, Cristina García-Vivar, Francine DeMontigny, Lucila Castanheira Nascimento

**Affiliations:** 1 Universidade de São Paulo, Escola de Enfermagem de Ribeirão Preto, PAHO/WHO Collaborating Centre for Nursing Research Development, Ribeirão Preto, SP, Brazil.; 2 Scholarship holder at the Coordenação de Aperfeiçoamento de Pessoal de Nível Superior (CAPES), Brazil.; 3 Universidad Pública de Navarra, Departamento de Ciencias de la Salud, Pamplona, Navarra, Spain.; 4 Université du Québec en Outaouais, Department of Nursing, Gatineau, Quebec, Canada.; 5 Scholarship holder at the Conselho Nacional de Desenvolvimento Científico e Tecnológico (CNPq), Brazil.

**Keywords:** Qualitative Research, Family Nursing, Pediatric Nursing, Family, Hope, Chronic Disease, Investigación Cualitativa, Enfermería de la Familia, Enfermería Pediátrica, Familia, Esperanza, Enfermedad Crónica, Pesquisa Qualitativa, Enfermagem Familiar, Enfermagem Pediátrica, Família, Esperança, Doença Crônica

## Abstract

**Objective:**

to analyze narratives about the experience of hope of families in the context of pediatric chronic illness.

**Method:**

a narrative research using Family Systems Nursing as a conceptual framework. Three families of children and adolescents diagnosed with complex chronic illness participated in this study, totaling 10 participants. Data collection was developed using family photo-elicitation interviews. Family narratives were constructed and analyzed according to inductive thematic analysis with theoretical data triangulation.

**Results:**

the analytical theme - *Waves of Family Hope in the Context of Pediatric Chronic Illness* - is composed of four different types of hope: uncertain hope, caring hope, latent hope, and expectant hope. Movement through these hopes generates a driving energy and depends on a number of factors: support, information, searching for normality, and thoughts and comparisons.

**Conclusion:**

the results highlight the interaction and reciprocities of the members of the family unit, and the dynamics of hope, and illustrate the different types of hope and the factors that influence them. This study highlights the experience of hope as a family resource rather than just an individual resource, and supports health professionals in the planning of family care considering hope as an essential and dynamic family resource.

## Abstract (es)

**Objetivo:**

analizar las narrativas sobre la experiencia de esperanza de las familias en el contexto de la enfermedad crónica pediátrica.

**Método:**

investigación narrativa que utilizó la Enfermería de Sistemas Familiares como marco conceptual. En este estudio participaron tres familias de niños y adolescentes diagnosticados con enfermedades crónicas complejas, con un total de 10 participantes. La recogida de datos se desarrolló mediante entrevistas familiares con foto-elicitación. Se construyeron y analizaron narrativas familiares siguiendo el análisis temático inductivo con triangulación teórica de los de datos.

**Resultados:**

el tema analítico - Olas de esperanza familiar en el contexto de la enfermedad crónica pediátrica - se compone de cuatro tipos diferentes de esperanza: esperanza incierta, esperanza cuidadora, esperanza latente y esperanza expectante. El movimiento a través de estas esperanzas genera una fuerza motriz y dependiente de varios factores: apoyo, información, búsqueda de la normalidad, pensamientos y comparaciones.

**Conclusión:**

los resultados destacan la interacción y reciprocidad de los miembros de la unidad familiar, la dinámica de la esperanza, e ilustran los diferentes tipos de esperanza y los factores que los influyen. Este estudio destaca la experiencia de la esperanza como un recurso familiar, en vez de un recurso individual, y apoya a los profesionales de la salud en la planificación del cuidado familiar considerando la esperanza como un recurso familiar esencial y dinámico.

## Abstract (pt)

**Objetivo:**

analisar as narrativas de famílias sobre suas experiências de esperança no contexto da doença crônica pediátrica.

**Método:**

pesquisa narrativa, que utilizou a teoria de sistemas familiares aplicada à enfermagem como referencial teórico. Compuseram a amostra deste estudo três famílias de crianças e adolescentes diagnosticados com doenças crônicas complexas, em um total de 10 participantes. Os dados coletados por meio de entrevistas com foto-elicitação permitiram a construção de narrativas familiares, as quais foram analisadas de acordo com a análise temática indutiva e triangulação teórica dos dados.

**Resultados:**

o tema analítico - Ondas de esperança familiar no contexto da doença crônica pediátrica - está composto por quatro diferentes tipos: esperança incerta, esperança cuidativa, esperança latente e esperança expectante. O movimento suscitado por essas esperanças gera uma energia motriz e depende de uma série de fatores, tais como apoio, informação, busca pela normalidade, pensamentos e comparações.

**Conclusão:**

os resultados evidenciam a interação e reciprocidades dos membros da unidade familiar, bem como a dinâmica da esperança, os diferentes tipos de esperança e os fatores que os influenciam. Este estudo destaca a experiência de esperança como um recurso familiar e não apenas individual, além de auxiliar os profissionais de saúde no planejamento do cuidado familiar considerando a esperança como um recurso familiar essencial e dinâmico.

## Introduction

In pediatrics, complex chronic health conditions include chronic illnesses and infectious diseases^(1)^. They are characterized by long duration and continuous care. During chronic illnesses, there are periods of clinical instability, which can lead to hospitalizations and complex care. Usually, there is no cure and children and adolescents need continuous health monitoring and support^(1)^. Due to this, the diagnosis of a pediatric chronic illness impacts all family members, which alters family relationships and dynamics^(2-3)^. The diagnosis affects children and adolescents on physical, emotional, and psychosocial levels^(4-5)^. Therefore, families recognize that the disease requires changes in routine and responsibilities^(6)^. In this context, the family needs to adapt, and hope is one of the resources used in times of crisis^(4,7-8)^.

Hope is an inherent resource of human experiences^(9)^. Several authors have explored the concept of hope, in different contexts and from different perspectives^(8,10-12)^. From the point of view of psychology, hope may be related to resilience^(13)^. However, as it is a complex concept, there is no common definition to be applied universally^(9,14)^. In the pediatric context, recently a thematic synthesis of qualitative studies identified the experience of hope in families living with pediatric chronic illness^(15)^. The review included 31 studies, and evidenced hope as a family resource. Family hope is dynamic, and its changes depend on the family’s experiences with chronic illness, time, and relationships between family members. Unlike other studies, the review highlighted the connectivity of hope among family members, and how they seek to balance it^(15)^.

In addition, the review highlighted that most of the included studies presented only one family member’s perspective on hope. It was recommended for future qualitative studies to: include the family unit; identify the experience of hope in different types of chronic conditions; and use iterative data collection to explore deeper children’s and adolescents’ narratives^(15)^. Alongside these knowledge gaps, research priorities in pediatric nursing have indicated the need for studies with families, which include the perspectives of several members. Also, there is a need for research on long-term illness, family-centered care, the impact of the illness on families, and resources that support the family in the context of the illness^(16-17)^. Hence, the question for this study was: How do families in the context of pediatric chronic illness experience hope? This study aimed to analyze narratives about the experience of hope of families in the context of pediatric chronic illness.

### Conceptual framework

This study adopted the conceptual framework of Family Systems Nursing^(18-19)^. This framework highlights that the family is a care unit, and that the family system is a part of a larger suprasystem and is composed of several subsystems. The family unit is greater than the sum of its parts - that is, its individual members. When a family member is affected (with a diagnosis of a chronic illness, for example) its members are also affected to varying degrees^(19)^. Family organization and functioning are altered, and family members seek a balance between change and stability. The family balance is in the coexistence between change and stability in the different phases of the life cycle^(19)^.

Families studies that use this theory are able to explain individual and family functioning through observation and analysis of family interaction and how they use the resources available to achieve a family goal^(19)^. Because of the recognition and disseminated use of this theory both in practice and in research, this study uses Family Systems Nursing as the conceptual framework to explore how the family unit uses the resource of hope.

## Method

### Type

This is a narrative research^(20)^ which used family photo-elicitation interviews^(21)^. Narrative research is a complex and dynamic method that provides an overview of research based on constructing and interpreting narratives as stories of experience. We chose Squire’s (2013) experience-centered narrative approach to explore the phenomena of this study. These types of narratives involve movements, successions, progressions, or sequences, usually temporal, and are connected with a significant fact, which is a changing point in the narrator’s life - for example, a diagnosis of chronic illness^(20)^. In the present study, narratives were structured as follows: introduction, development and conclusion. They contained elements such as characters, time, space, environment and plot. The process of narrating introduced the representation of the *I*, because everything that was told had a meaning. Therefore, this method aimed to understand the experience of individuals through the reports of experienced events and the co-constructed narratives that were stories and statements constructed from dialogues between the participants^(20)^.

### Scenario

A convenience sample of participants was recruited face-to-face in 2019, when the children or adolescents were hospitalized in the pediatric wards of a public university hospital, located in the interior of the state of São Paulo, Brazil.

### Population

Children and adolescents diagnosed with complex chronic illness and their families were invited to participate in the research. In this study we considered “family members” as all people that children and adolescents described as such^(19)^. Considering the experience of the diagnosis, children and adolescents were excluded if they had been newly diagnosed (less than six months). Family members who did not actively participate in the care or daily routines of the child or adolescent were excluded, as were family members under eight years old.

To participate in this study, at least one family dyad was included. The number of family members interviewed varied according to their availability. The determination of the number of participants included in this study and the interruption of recruitment occurred when the data collected were sufficient to achieve the aim proposed^(22)^. This was possible because of the exhaustive data analysis conducted concurrently with data collection, which provided an in-depth analysis of the data of each participant and of the families, as well as the similarities and uniqueness of the experience among them.

### Data collection

**Figure 1:**
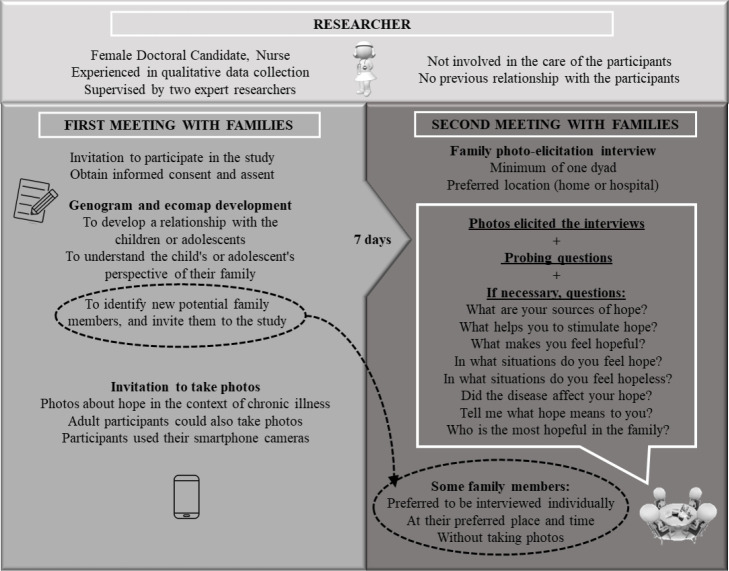
Process of data collection

In the pediatric hospital in 2019, families were personally invited to participate in the study. In this first meeting, after explaining about the study and obtaining the written consent/assent, a female researcher built a genogram and ecomap of the family with the child or adolescent. The guiding question was: Tell me about who is your family? In the end of first meeting, the researcher invited the family to take photos about their perspective of hope, using their own smartphone cameras. The photos were used in the second meeting to perform a family photo-elicitation interview^(21)^. This method of data collection elicited narratives and promoted reflection of the family experiences^(21)^. When necessary, questions (see Figure 1) were used to deepen data collection. Some family members preferred individual interviews, without using their own photos to elicit the narratives (Father, Family A and Aunt, Family B). At least two meetings with each family were held, each meeting lasting about an hour and a half. The detailed data collection process is illustrated in Figure 1.

### Data analysis

Inductive thematic data analysis was used to analyze family narratives, and the following phases were performed^(23)^: (I) Familiarization of data from repeated readings of the transcribed interviews. Subsequently, narratives of each family were constructed. Each family narrative was composed of family and/or individual interviews. Family narratives were structured with introduction, development, and conclusion, and they contained elements such as characters, time, space, environment, and plot. (II) Initial codes were identified inductively from the narratives. The QDA Miner Lite^®^, qualitative data analysis software, was used in this stage; (III) From the similarities and differences between the initial codes, a narrative synthesis was constructed inductively. In this step, it was possible to identify the different types of hope, the factors that influence them, and the similarities and uniquenesses in the families’ experiences. (IV) An analytical theme was developed inductively from the narrative synthesis, according to the conceptual framework chosen. The analytical theme was also constructed based on theoretical data triangulation^(24)^, with the results from the thematic synthesis of qualitative studies about hope^(15)^; (V) The analytical theme was named and refined.; (VI) The results were constructed, presenting the analytical theme: “Waves of Family Hope in the Context of Pediatric Chronic Illness”. The analytical steps were performed by the first author and discussed and validated by the three other authors, who are experts in this type of analysis. The photos elicited the participants’ narratives, but these images were not analyzed, only their narratives. The set of families’ narratives is 55 pages long. The family genogram and ecomap helped the researchers to contextualize the data and to better understand the family structure and dynamics. Field notes helped researchers reflect on the data. Transcripts and analyses were not shared with participants.

### Ethical issues

This study was approved by the ethics committees (Ethical Approval Numbers: 2.902.779: 2.902.779; 2.911.296; 9146418.7.0000.5393). The written consent of adult participants was obtained. Children and adolescents affirmed their desire to participate in the study by signing the Assent Form when a responsible adult allowed their participation.

### Rigor

The rigor of this study was ensured by^(25-26)^:

- Credibility - rigorous data analysis developed by a research team, supported by the quotes that illustrate the findings and audit trail.
- Transferability - presentation of families’ sociodemographic data.
- Reliability - detailed description of the method, following the Consolidated Criteria for Reporting Qualitative Research (COREQ)^(27)^.
- Confirmability - presentation of the limitations and strengths of the study and by the researchers’ reflexivity.

## Results

**Figure 2:**
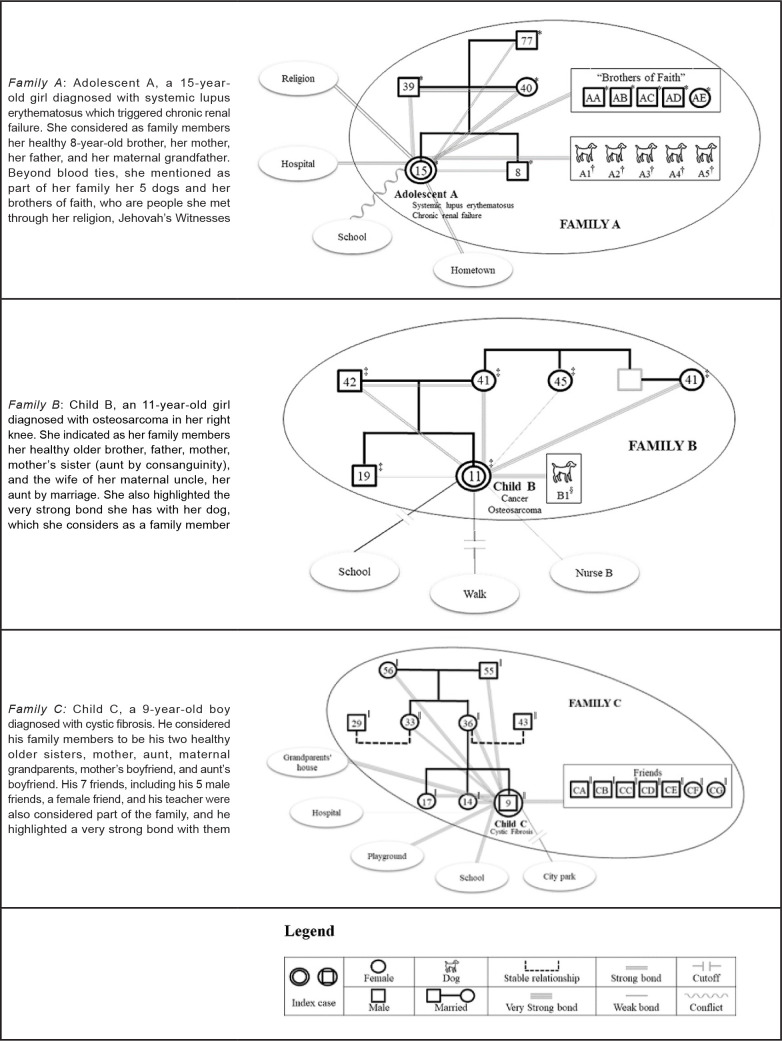
Description of family characteristics and their genograms and ecomaps. Ribeirão Preto, SP, Brazil, 2019 *77, *39, *40, *15, *8, *AA, *AB, *AC, *AD, *AE = Family members of Family A;^†^A1,^†^A2,^†^A3,^†^A4,^†^A5 = Dogs of Family A;^‡^42,^‡^41,^‡^45,^‡^41,^‡^19,^‡^11 = Family member of Family B;^§^B1 = Dog of Family B;^||^56,^||^55,^||^29,^||^33,^||^36,^||^43,^||^17,^||^14,^||^9,^||^CA,^||^CB,^||^CC,^||^CD,^||^CE,^||^CF,^||^CG = Family members of Family C

This study included 10 participants from three families - Family A: Adolescent A, Mother and Father (n=3); Family B: Child B, Mother, Father and Aunt (n=4); Family C: Child C, Mother and Sister (n=3). Only one family refused to participate as they did not want to bring up memories of the treatment. Figure 2 shows in detail the description of family characteristics and their genograms and ecomaps.

### Waves of Family Hope in the Context of Pediatric Chronic Illness

**Figure 3:**
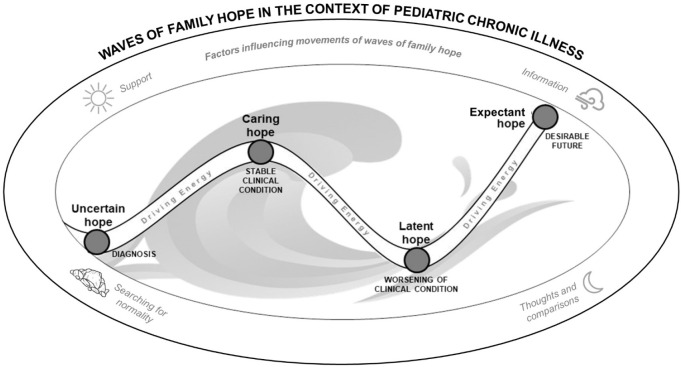
Waves of Family Hope in the Context of Pediatric Chronic Illness

The analytical theme is presented through a metaphor with ocean waves, which we entitled: *Waves of Family Hope in the Context of Pediatric Chronic Illness* (Figure 3). The family unit is represented by the wave, and the dynamics of family hope by its movement. There are different types of ocean waves; depending on their movement, they are able to propel, immerse, or keep people afloat, representing the same parallel of *waves of family hope*. The characteristics of each wave represent the individuality of the experience of each family.

Waves of family hope are composed of four different types of hope: uncertain hope, caring hope, latent hope, and expectant hope. The movement through these types of hope depends on factors such as: support, information, searching for normality, and thoughts and comparisons. In Figure 3, these factors are compared to those that influence the formation and movement of waves: sun, wind, seabed, and moon. Therefore, the waves of family hope are the result of the interaction of family members (their relationship, roles, alliances, power, affiliations and cohesion), time (past, present and future related to lived and expected experiences), and context (such as cultural context and type of chronic illness) which present factors that influence their dynamics. Because the waves of hope are dynamic, they will not always be composed of the 4 types of hope, or they will be experienced by the family in a longitudinal way. The waves of hope are forming and breaking constantly.

The waves of family hope in the longitudinal experience of chronic illness begin with the diagnosis. At this moment, the family experiences *uncertain hope*. The rise in family hope occurs over time. When the child reaches a stable clinical condition during treatment, the family experiences *caring hope. Latent hope* occurs when there is a worsening of the child’s clinical condition, and the family feels that hope has been lost, even though it is still present without manifesting. *Expectant hope* is experienced by the family as they project a desired future, in which the only remaining resource is hope.

The waves always generate energy due to their driving movement. In our results, this energy represents a family member who is able to drive energy through wave movements to propel family hope. Wave formations and movements occur constantly. Due to this dynamic process, families experience at different times the different types of hope throughout the illness trajectory.

**Figure 4:**
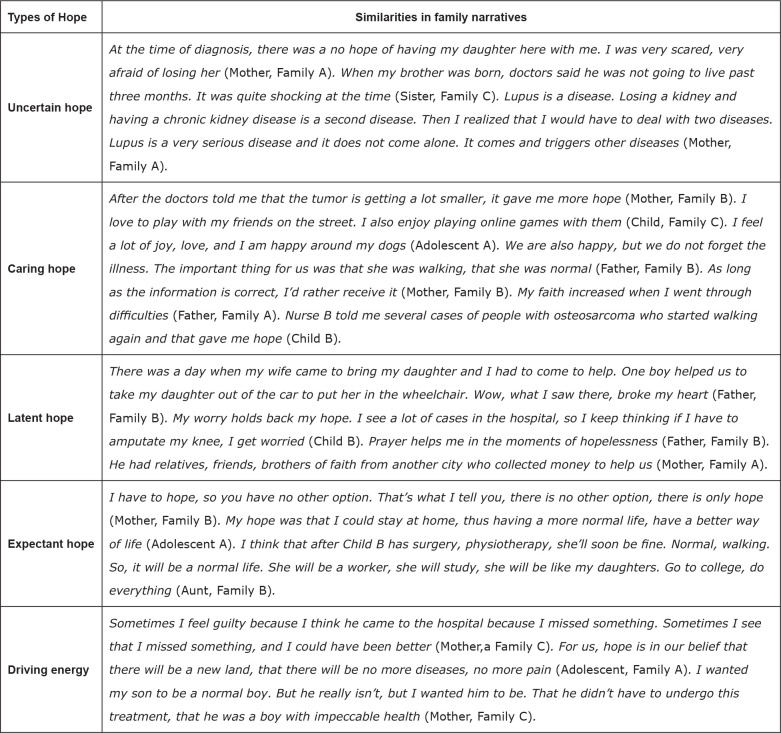
Similarities in family narratives (n=10) according to types of hope. Ribeirão Preto, SP, Brazil, 2019

**Figure 5:**
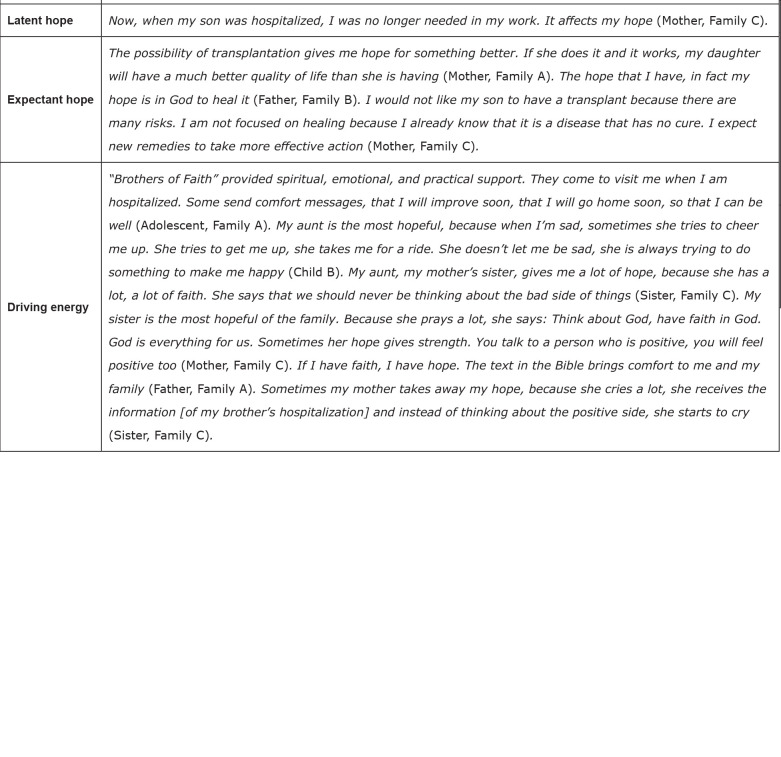
Uniqueness in family narratives (n=10) according to types of hope. Ribeirão Preto, SP, Brazil, 2019

The types of hope identified in this study are described below. Figure 4 presents the similarities between the narratives of types of hope among the families. Figure 5 shows the unique experiences within the family related to the types of hope.

#### Uncertain hope

At diagnosis, families begin a wave of experiences and emotions. Hope seems invisible because uncertainty prevails. Therefore, families experience *uncertain hope*, which is related to fear of the unknown, fear of death, suffering, and emotional shock. Family members try to protect the child from *uncertain hope* by hiding emotions and avoiding talking about the seriousness of the situation. *I didn’t even cry during the diagnosis consultation. But it is so difficult. My daughter cried. If I cry in front of her it seems that it gets even more complicated. I was holding on, so as not to show that the situation is so serious* (Father, Family B).

*Uncertain hope* was evidenced by questions about the cause of the illness and the family’s difficulty in accepting the diagnosis. Uncertainty was also related to the type of diagnosis. Diagnosis of cancer has a stigma, although it is the only chronic illness included in this study with the possibility of a cure, and cystic fibrosis is associated with early death. To reduce uncertainty, families need to obtain information about the diagnosis. However, this did not always help to promote family hope, but in fact reinforced the thoughts of uncertainty, especially when the information was related to negative outcomes, such as death.

Gradually, after the diagnosis, family members reorganized their responsibilities and family plans to take care of the child. The family looked for situations and factors that could propel family hope, so that they could move on from uncertainty. However, the maintenance or return to *uncertain hope* was related to the possibility or discovery of new diagnoses.

#### Caring hope

Families experienced *caring hope* when children had a stable clinical condition. The possibility of an effective treatment, and positive impact of the treatment on the child’s clinical condition helped to propel and maintain the wave of *caring hope. Caring hope* was also related to the decrease in the length of the child’s hospitalization and their well-being and happiness. *It gives me a lot of joy and happiness to know that my brother doesn’t need to be hospitalized. Because the other children with cystic fibrosis practically live in the hospital* (Sister, Family C).

This context allowed children to maintain a positive perspective and *feel normal*, as well as their peers, when they performed daily activities and forgot about their treatment and diagnosis. As a daily activity, all children mentioned playing with dogs. For them dogs could promote hope by providing happiness and a feeling of normality. Child C expressed a desire to have a dog, but his mother would not allow it because she thought it would hinder his treatment. Other family members evidenced the positive impact that dogs had on the child’s and family’s well-being. The exception was the father of Family A, who believed that the maintenance of *caring hope* come from faith.

Family members did not have the same feeling of normality as children. The responsibility for care, signs of illness, and symptoms and concerns about episodes of urgency or worsening of the clinical condition constantly reminded them of the fragility of the child’s health and the dynamism of family hope. Upon receiving clear, empathetic, and realistic information about treatment, families felt more optimistic. Support for religious beliefs and faith were also factors that maintained the family’s optimistic outlook and helped them cope with treatment. In addition, the families wished to have similar experiences with good outcomes, to have positive thoughts, and consolidate their *caring hope*.

#### Latent hope

Families experienced *latent hope* when the child’s clinical condition worsened. The decline in their optimistic perspective affected the movement of waves of family hope, and families felt as if they had lost hope. Invasive treatment, or situations that evidenced the child’s fragility, reinforced *latent hope. When she lost her kidney, she started hemodialysis, which is a very aggressive treatment. So, my hope was unsettled* (Mother, Family A).

*Latent hope* was also reinforced with thoughts about death and comparisons with bad outcomes. One of the families’ lack of financial support was a factor that influenced the maintenance of *latent hope*. Families considered the hospital context to be a constant reminder of the child’s health condition. During hospitalizations, it was difficult for families to maintain optimistic thoughts, or to distance themselves from negative cases or information about the illness. In an attempt to protect themselves from *latent hope*, families tried to neutralize thoughts about worries and death, and avoid people who would make negative comments about the child’s condition.

The resources used by families to let go of *latent hope* were faith, and support among family members. Beliefs arising from faith in the divine brought comfort in times of crisis, and a more optimistic view of the situation. The emotional, financial, and instrumental support among family members helped to cope with the crisis and search for the propulsion of family hope.

#### Expectant hope

When a family’s resources to maintain family hope were depleted, what remained was *expectant hope*, which was directed towards the future. For the near future, families expected a daily routine without the need for treatment, or, at least, a decrease in the complexity or amount of care. Families’ plans for the future were affected by the illness and treatment. Therefore, families replanned their dreams and started to value the small things in life. *I had a lot of dreams and after the diagnosis I lost my dreams. After the diagnosis everything changed in my life. Everything was normal. Before I did not value small things, today I do* (Child B).

The future projected by families with expectant hope envisioned the health of the child - as well as of the other healthy members of the family, or as being the same as it was prior to the illness. Also, in this desirable future, children would live a *normal* life just like their healthy family members; they would study, work, and be happy. However, families knew that the reality of the future depended on the type of treatment available.

For Family A, a kidney transplantation would be a source of hope, but it would not bring the cure, because the need for treatment would remain. Family B wanted the cure for cancer, and they redirected their lives and hopes towards reaching this possibility. Family C believed that lung transplantation would not be the best treatment; however, research about new medications could offer more effective treatment. They desired a long, high-quality life for Child C. The dynamics of expectant hope were related to changes in the child’s clinical condition, which led to new perspectives for the future, or a return to other types of hope.

#### Driving energy

The waves of hope are dynamic and generate *driving energy*. This is represented by a hopeful person in the family, who is able to promote family hope in times of crisis. Family members reorganized their responsibilities and family plans to take care of the child. Mothers assumed the responsibility of being the primary caregiver of the child. Although mothers were hopeful, they felt overburdened with the primary caregiving responsibilities. Due to the responsibility of care, mothers also felt guilty during periods of worsening of the child’s clinical condition. Given this scenario, mothers were not able to be the family reference for the responsibility of promoting the *driving energy* of family hope.

All family members were able to identify the person who could *drive the energy* of family hope in times of crisis. This was accomplished through speeches and optimistic thoughts, faith in empowering beliefs, prayer, emotional support, financial support, visits, and playing with the child. Each family identified the *driving energy* person: for Family A, it was the *brothers of faith*; for Family B, the aunt; and for Family C, also the aunt. These persons had a strong emotional bond with family members, but they did not have the responsibility of daily care, which led them to have a more optimistic perspective. *“Brothers of Faith” are always on hand to help us. They have always supported us, through the words of God. This strengthens me. They also help me with money. They were with open arms to help us* (Father, Family A).

Although Family B’s aunt believed she could influence family hope, she did not identify her hope as being influential. Some family members identified who was able to interrupt the propulsion of waves of family hope and, consequently, the driving energy. For the members of Family A, their religious beliefs protected them from the influence of others’ hopelessness. In Family B, Child B identified his brother as the promoter of her hopelessness. Sister C believed that her mother promotes hopelessness in the family. *My brother takes away my hope. He and I fought a lot, he is very sincere. Then one day we were talking, that I forgot to take the capsule, my medicine in the morning. He said something that hurt me: If you continue like this, you will lose your leg! It takes away hope* (Child B).

## Discussion

This study enabled us to identify the narratives about the experience of hope of families of children and adolescents living with chronic illness. The results showed that the illness impacts family members differently throughout the chronicity process. However, individual hope developed into a family resource due to connectivity between family members. Family processes are based on interactions between family members, who support each other, share affection and communicate. In this process there can also be conflicts, so families look to their strengths to face challenges and crises^(28-29)^. Our results demonstrate that families used family hope to maintain a positive perspective. Emotions, behaviors, and positive thoughts from adult relatives, especially parents, generate security, emotional regulation, and less suffering for children, and in addition restore hope^(9,28)^.

The results showed the dynamics of family hope through the experience of different types of hope during the process of chronicity. Hope is a resource with many faces, that is always present, even if it its presence is not consciously registered^(9)^. This characteristic appeared in the study results and was related to the family crises being experienced, such as the moment of diagnosis and worsening of the child’s clinical condition, represented by uncertain hope and latent hope.

The feeling of uncertainty is usually present in the families of children and adolescents living with chronic illness. A study developed with parents in the context of pediatric palliative care showed that their experience of hope was based on uncertainty^(30)^. The uncertainty was related to the concern about the worsening of the child’s health, and his death, leading to an abrupt loss of hope^(30)^. However, another study showed that hope was a resource used by parents in anticipatory grief, which is the feeling of grief that occurs before an imminent death. Even after the child’s death, parental hope was present through the belief that the child would be in a better place, or that one day the parents would find them again^(31)^. Differently from the study mentioned before, family hope was promoted by avoiding thinking about the possibility of the child’s death. Our results also demonstrated that uncertainty was the feeling that promoted uncertain hope and was mainly related to the moment of diagnosis.

Corroborating our results, hope is considered by parents of children and adolescents with chronic diseases as the first and last strategy for dealing with moments of crisis^(30)^. We highlighted that types of hope act as waves of family hope, changing in the face of different factors and unique family experiences. Other studies also presented the perspective of different types of hope, highlighting this process as a constant metamorphosis^(9,30)^. The factors that influence hope change according to the context in which it is experienced^(32)^. Our study showed four factors that influenced family hope. Further research should investigate how these factors act in hope, highlighting those that can be protective.

The results of this study demonstrate that information can promote or decrease family hope. Therefore, families need to receive information, and it is the responsibility of the healthcare team to inform them in a clear and empathetic way, emphasizing that information can change according to the child’s therapeutic plan and ongoing clinical condition^(33)^. Health professionals help to promote family hope by giving information in a clear and empathetic way, while omitting information or lacking empathy during communication can decrease family hope. This process can affect the families’ well-being^(34)^. For families, thoughts and comparisons can bring a positive or negative perspective to the situation. Studies indicate that when there is a positive perspective due to thoughts and comparisons, there is restoration of hope and family functioning^(9,28)^. Thoughts or comparisons that generate a negative perspectives are able to decrease family hope^(15)^.

With regard to support, the presence of this factor was able to promote or maintain family hope throughout the chronicity process. This factor was related to the connection between family members and beliefs, such as faith in the divine. In the country where the participants of this study are from, most people are Christians, Catholics or Protestants affiliated with some religion^(35)^. However, in this context, there is an increase in evangelicalism^(35-36)^. The country’s social, cultural, and historical issues, mainly related to colonization and immigration, affected the importance of religion in people’s lives. At the same time, the population experiences a plural spiritual identity^(36)^.

Metamorphic spiritual identity is like a mosaic of beliefs in Brazil. The population’s spiritual identity is diverse and essential in the lives of those who experience it, hindering the accuracy of official census data on this topic^(36)^. Given this context, health professionals need to identify and stimulate the family source of support, and consequently, family hope may be promoted. Nurses and other health professionals can initiate interventions to identify family strengths that help families express their feelings and beliefs related to their cultural context^(37-38)^.

In our results, families sought normality through activities that generated the feeling that the child would not be sick anymore. This search was related to the moments in the child’s clinical condition - caring hope - or in the projection of the ideal future - expectant hope. The lack of this feeling was exacerbated in uncertain hope and latent hope. Hope is an indispensable resource, and acts as the vital force for parents^(30)^. It anticipates that the future will bring better possibilities than the past and the present^(9)^. Among these mysterious possibilities is a sense of normality, which is usually linked to a cure, that can be associated with a miracle^(39)^. For the families in this study, the ideal future would not have moments of crisis related to the illness. It would be like stepping out of the dynamics of waves of family hope, through its propelling movement, to achieve the safety of the shore.

Due to the dynamics of the waves of family hope, the search for moments of full hope is constant. However, achieving this is a fragile process, as the context and factors that led the family to that moment can change abruptly, starting a new wave. Despite this, our study contributes to a new perspective on the influence of family members on family hope. The waves of family hope generate driving energy, which can be illustrated by a hopeful family member who is able to promote or maintain family hope. Hope is recognized as a unitary human experience^(9)^. However, when presenting hope as a family resource, care for the family can be planned, considering it as a systemic unit. By changing the perspective, nurses and other health professionals will be able to identify and evaluate hope through the lens of the family system and their unique experiences. Family Systems Nursing theory acknowledges that *illness is a family affair* ^(19)^; with our study, we extend that acknowledgement by saying that *hope is also a family affair*.

This study has strengths and limitations. We consider as its strengths: a) the inclusion of the family unit^(21)^; b) the conducting of family photo-elicitation interviews which allowed for iterative data collection to obtain family members’, children’s, and adolescents’ narratives^(21)^; c) the rigor used in the development of this study and the detailed description of the method, which may serve as a model for future qualitative research; and d) the use of a theoretical framework and theoretical data triangulation, which allowed for a deeper data analysis. As a limitation, we highlight the inclusion of a homogeneous sample, due to the small variability in family structures, the context of living, and diagnosis of chronic illness. However, the inclusion of different complex chronic diseases as well as the deepening of the experience of hope in each family has contributed to new knowledge about the characteristics of hope in these families, which can guide future qualitative studies.

The results of this study can help health teams to plan a systemic family care while considering hope as an essential and dynamic family resource. Our results contribute with the proposal of the *Waves of Family Hope*, explaining how family hope is dynamic in relation to the context, time, and family structure. In the educational field, this perspective can be used to teach nursing students to consider the aspects mentioned above in family-centered care. We emphasize that this study presents similarities and uniquenesses of the experience of hope of families in the context of pediatric chronic illness and the transferability and interpretation of its results need to consider the context in which it was developed.

With regard to future research, this study highlights the need to develop studies with different family structures, and in different cultural and care contexts, such as with families from eastern cultures, or in the context of pediatric end-of-life care. Also, there is a need to develop studies with health professionals, especially with nurses, to identify their perspectives about family hope, and the barriers they come across and the strategies they use to overcome them.

## Conclusion

This study analyzed the narratives about the experience of hope of families in the context of pediatric chronic illness and contributed to the perspective of *Waves of Family Hope*. The results corroborated with the theoretical framework and theoretical data triangulation used, which highlight the interaction and reciprocities of the members of the family unit, and the dynamics of hope. The family narratives allowed us to go deeper into the experience of family hope in the trajectory of chronic illness, and reveal evidence that it was composed of different types of hope and that its dynamics were influenced by four factors. Moreover, the movements of waves of family hope generated a *driving energy* that is able to promote family hope in times of crisis. These results can help health team to plan family care considering hope as an essential and dynamic family resource.
